# Effectiveness of Lighting Conditions on Shade Matching Accuracy Among Dental Students

**DOI:** 10.3390/dj13030130

**Published:** 2025-03-14

**Authors:** Christina Perou, Andrianos Petalas, Michaella Stoupi, Christina Hadjichristou

**Affiliations:** Department of Dentistry, School of Dentistry, European University Cyprus, 2404 Nicosia, Cyprus; cp202128@students.euc.ac.cy (C.P.); ap201193@students.euc.ac.cy (A.P.); ms201215@students.euc.ac.cy (M.S.)

**Keywords:** color, shade matching, prosthodontics, spectrophotometer, smile lite, artificial light source

## Abstract

**Background**: In prosthodontics, achieving esthetic success requires precise shade matching of restorations to natural teeth. This study evaluated the shade-matching abilities of fourth-year dental students using different tools and conditions in laboratory (LB) and clinical (CL) settings. **Methods**: In the LB setting, students matched blinded shade tabs to the VITA classical shade guide under natural daylight (ND), artificial light (AL), and a polarized filter (PF). In the CL setting, they determined the shades of patients’ central incisors using the same conditions. Participants also completed a questionnaire about their experience. **Results**: Quantitative analysis showed better shade matching in the LB setting, particularly with the PF (52% correct), compared to ND (50%) and AL (43%). In the CL setting, overall accuracy dropped to 32% across all conditions. No results were statistically significant. Qualitative feedback revealed that students found the spectrophotometer to have improved their accuracy and reliability compared to the ND and AL conditions. **Conclusions**: This study highlights the importance of incorporating technology as a validation tool in dental practice. Leveraging tools like spectrophotometers may enhance shade-matching accuracy, streamline processes, and improve patient satisfaction while balancing traditional methods with technological advancements.

## 1. Introduction

The shade of dental restorations is an important parameter for final patient acceptance [1]. Precise shade selection has become critical to the esthetic needs of patients [1,2]. Shade matching is an essential aspect of restorative, esthetic, and prosthetic dentistry and is considered one of the most important and difficult steps [3]. All the energy put into treatment planning, accurate tooth preparation, shaping, and surface texture is highly diminished if the patient is not satisfied with the color and translucency of the tooth at the end of treatment [3,4]. Tooth color consists of layers of enamel and dentin, which interact with light in ways that affect its appearance. The quality of light is critical in achieving accurate shade matching, as variations in lighting can alter how colors are perceived. Therefore, reproducible and correct color matching requires proper lighting and spectral dispersion [5]. Shade matching can be carried out using two methods: instrumental (with color measuring instruments) or visual (with shade guides) [2,3,6,7]. In the latter method, the dentist compares the patient’s tooth with a standardized shade, and this is the most commonly used method of shade determination in clinical dentistry [3,4,7]. However, various (subjective and physical) factors can influence or distort shade matching, such as eye fatigue, age, clinical experience, color deficiencies, judgment, mood, emotional fluctuations, illusions, and color blindness [3,7]. It also depends on the lighting conditions (intensity of the light source), the color of the walls, and the clothing of the patient and the staff [3]. Gender has also been shown to play an important role in shade matching, implying that females may achieve better results than males [3]. The color of natural teeth results from the combination of light reflected from the surface of the enamel and light scattered and reflected from both the enamel and dentine, the latter being the main source of color and modified by the translucency and thickness of the enamel [8]. Since this method relies solely on the human eye to determine tooth color, it leads to unstable and unreliable results [6].

While the human brain can distinguish around one million shades, advanced tools have been developed that can differentiate up to one hundred million shades. The natural variability in tooth color is extensive—electronic instruments can identify over 100,000 dental shades, whereas the human eye can distinguish only about 1% of them [9]. The most commonly used method for visual shade matching that is cost-effective is employing dental shade guides, which serve as standardized color references. Several shade guides are available based on hue, chroma, and value [7]. The easiest to understand of these three factors is the value, which corresponds to the lightness or darkness of a color, with a high value translating as white and a low value as black. Further, hue is the property that distinguishes one color family from another and is determined by wavelength. Finally, chroma is the most elusive property and indicates how saturated a color is. This property distinguishes a strong color from a weak color, so it is about the intensity of the color. Chroma is indicated by increasing saturation and correspondingly increasing numbers. Luminosity is defined by a sequence from lower-luminosity existing shades to the higher ones [7]. An example of a commercially available shade guide based on value is the VITA 3D-MASTER ^®^, and for hue, the VITA classical (Control) [10]. Shade matching is usually associated with homogeneously colored objects, but teeth vary in color and translucency; therefore, it is considered as a more difficult procedure.

The VITA classical shade guide, which was used for the completion of this research, offers precision and simplicity. It is a hue-based shade guide divided into four shade groups (A, B, C, and D), followed by the degree of saturation and color brightness [4,11,12,13,14]. The arrangement of the shades in the VITA classical is A1-A4 (reddish-brownish), B1-B4 (reddish-yellowish), C1-C4 (grayish shades), and D2-D4 (reddish-gray) [4].

Light is a necessary component for shade selection. To select the hue, the use of daylight light conditions is recommended, as natural daylight is the ideal setting. According to the Kelvin temperature scale, the color temperature of daylight with a clear sky and sun overhead is 5000–6500 K, while under a moderately overcast sky, this temperature is 6500–8000 K, and under a heavily overcast sky, it is 9000–10,000 K. The ideal conditions for tooth shade selection involve a light source with a color temperature of 5500–6500 K and a Color Rendering Index (CRI) of at least 90. Using multiple light sources can lead to metamerism, where the colors match differently under different lighting conditions [5,13].

The aim of this study was to evaluate the ability of fourth-year students to color match with different tools, such as the use of artificial light sources with a color temperature of 5500 K and the traditional method of shade matching under natural daylight in comparison to the spectrophotometer [1]. Another aspect of this study was the ability of biological females versus males in the process of shade selection.

Approval was obtained from the National Bioethics Committee (Cyprus National Bioethics Committee—CNBC).

The null hypotheses are as follows: (a) shade matching using light aids (artificial light—AL or polarized filter—PF) is more accurate than shade matching under natural daylight and (b) biological gender does not play an important role in shade matching.

## 2. Materials and Methods

For the conduction of this research, fifty (25 female and 25 male) 4th-year dental students at the European University of Cyprus participated. The curriculum of the undergraduate program at the School of Dentistry at our university spans a duration of five years, with the fourth year serving as the initial clinical year. During the second and third years of preclinical training, students are introduced to the theoretical principles of conventional shade matching using visual shade guides as a reference. Artificial light tools or spectrophotometers are not traditionally included in clinical shade-matching procedures. Students were evaluated for their color discrimination ability using the pseudoisochromatic plate (PIP) color vision test (Ishihara test) and those that participated presented negative color blindness [1,10]. The location of the experiment was the EUC Dental Clinic, in two different stations, one with windows for the natural daylight condition and another without windows for the lighting conditions with artificial light and the polarized filter. The ceiling of the clinic was white, whereas the walls were light gray [15].

**Figure 1:**
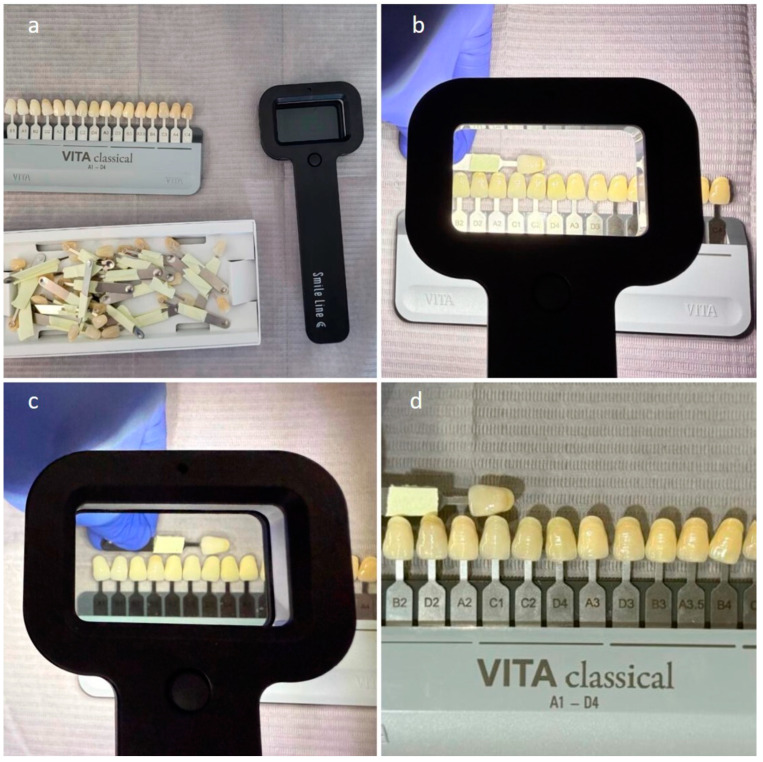
Set up of the laboratory part (LB) of the experiment. (**a**) Shade matching under natural daylight (**b**), under artificial light (**c**), and using the polarized filter (**d**).

The procedure consisted of two phases, one laboratory phase (LB) and one clinical phase (CL). The first phase (LB) involved a color-matching exercise of shade tabs from a known company of dental shade guides under three lighting conditions: natural daylight (ND), artificial light with a polarized filter (PF), and artificial light (AL) (Figure 1). The materials required for this part were three shade guides (VITA classical), of which two were blinded and one unblinded. In addition, one artificial light of 5500 K (Smile Lite, Style Italiano) and one polarizing filter (Smile Lite, Style Italiano) were used [1]. All tabs were removed from the two shade guides and each tab’s code was blinded [12]. Students were asked to randomly select two shade tabs for each procedure (ND, PF, AL) from the blinded guides and compare them to the full, unblinded shade chart placed on a gray bib (neutral background) [10,13]. The shade tabs were arranged according to the value in the unblinded shade chart [14,16]. In total, each student used six blinded shade tabs during the LB phase and was given a total of six minutes for the procedure. Only one student was allowed in the test room at a time to prevent interference from the other students. The shade-matching procedure was conducted in the same room and under the same conditions for all students. If necessary, participants were asked to relax their eyes by looking at the gray background for a few seconds. According to the manufacturer’s instructions, the students were instructed to hold the SmileLite device 10 cm away from the tooth on which they were making the shade selection. Their shade selection was recorded by one of the researchers on a form. Upon completing the selection process, they were asked to leave the room [1].

**Figure 2:**
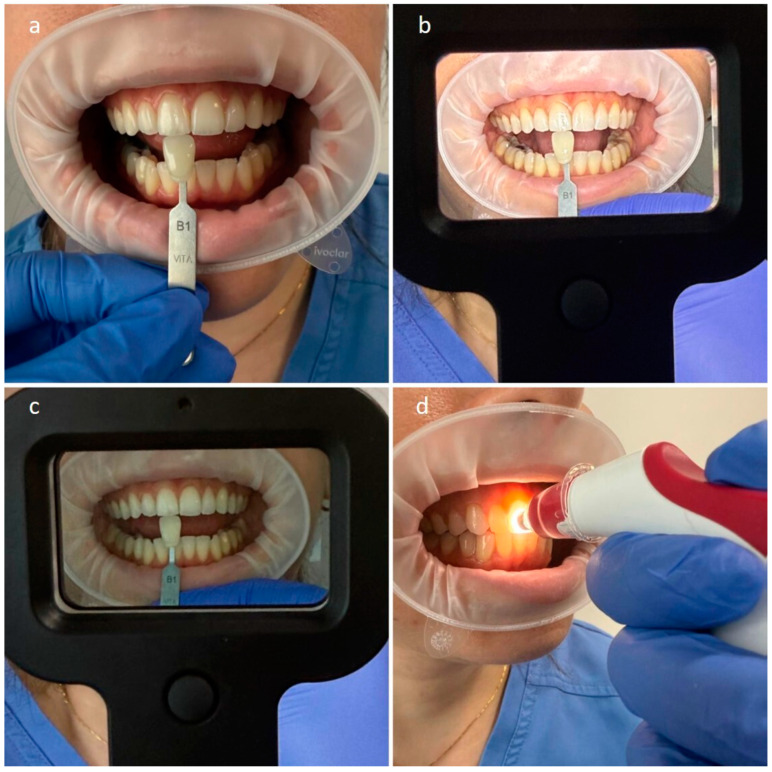
Set up of the clinical part (CL) of the experiment. Shade matching under natural daylight (**a**), under artificial light (**b**), using the polarized filter (**c**), and validation using the spectrophotometer (**d**). Retractors were used only for photographic purposes.

The second phase of the study involved a clinical (CL) exercise at the same stations as the LB (Figure 2). The teeth selected for shade matching were the maxillary central incisor (# 21—middle third), which had no restorations [1,8,11,17,18,19]. During the experiment, the selected tooth was polished using a rubber cup and a pumice stone for 10 s and then rinsed with water for 5 s [19].

The same participants from the LB part were paired for the CL part. Each participant performed the shade matching for the CL part on the central incisor of their partner. One participant assumed the role of the examiner, while the other acted as the patient, and vice versa. The patient was seated in a chair away from the windows and wore a gray bib. All patients were asked not to wear lipstick or make-up and to pull their hair back. If they wore glasses, they were removed. During the second part of the examination, both the patient and the examiner were seated at a 90-degree angle, with the latter’s eye line as parallel as possible to the patient’s occlusal plane and at an average distance of one arm’s length from the examiner. Otherwise, if teeth are observed more closely, they tend to appear larger and brighter [5,19].

The examiners placed the shade tabs edge to edge with the central incisor and matched a shade from the shade guide with the middle third of the labial surface of the selected tooth in ND. A time interval of 5 s to rest the eyes on a gray bib was necessary between the actual shade selections, as the dark oral cavity absorbs light and can distort shade selection [3,5,6]. The same procedure was repeated for the other two lighting conditions (AL, PL).

**Figure 3:**
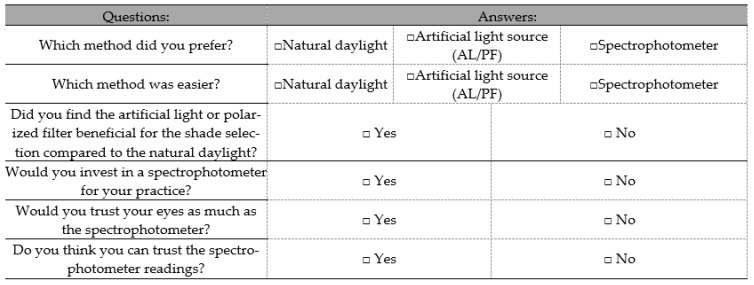
Questionnaire for the qualitative assessment.

During the last step of the examination, all participants carried out the shade matching using a spectrophotometer [20,21]. This measurement served as a comparative shade of the examined natural tooth. This procedure also served to evaluate whether spectrophotometers enhance students’ understanding and ability to accurately determine tooth shade. According to the manufacturer’s instructions, the spectrophotometer was calibrated for each subject by holding the 5 mm probe tip against a calibrated block until the device beeped. The measured tooth shade was accepted when three consecutive identical readings were obtained for each tooth [19]. For the instrumental shade measurement, the middle third of the labial surface of the maxillary central incisor was selected to best represent the tooth shade. Therefore, for each measurement, the instrument was set to “single tooth” mode, and the tip of the spectrophotometer was positioned at a 90° angle, with zero contact distance. The participants recorded their choices on a form before leaving the room [19,22,23]. After completing the two parts of the experiment, participants were asked to complete a questionnaire, the content of which is shown in Figure 3.

### Statistical Analysis

The data were statistically analyzed using SPSS software 25.0.0.0 (IBM Corp., Armonk, NY, USA). The variables analyzed were the three light conditions (ND, PF, AL), the LB and CL part, and the biological gender of the participants. The McNemar test was used to compare correct predictions under the three light conditions between male and female participants for the CL and LB parts. A t-test was applied to compare (i) the deviation of the participants’ prediction with the actual tab shade of the LB experiment, (ii) the natural tooth color of the CL experiment, and (iii) the possible differences in the mean absolute difference between male and female students. A *p*-value < 0.05 was considered statistically significant.

## 3. Results

### 3.1. Quantitative Results

#### 3.1.1. Laboratory Phase (LB): Shade Tab Predictions

For the LB part of this study, participants had to predict the shade of the selected tab according to the equivalent from the shade guide. The results in descending order were as follows: 52% of the participants correctly predicted the shade of the tab based on the PF, followed by 50% correct predictions in ND, and, finally, 43% correct predictions in AL. The color mapping between men and women was the same or almost the same in each condition. In natural light, participants of both sexes had 50% correct color predictions. With the PF, this percentage increased slightly to 52%. In AL, 42% of the male participants’ predictions and 44% of the females’ predictions were correct. The McNemar test was used to compare correct predictions in the LB condition between male and female students, and this showed that there were no significant differences in correct predictions in the LB condition between male and female students, at a 5% significance level (Table 1).

Of the fifty students who participated in the study, fifteen (30%) (eight male, seven female) had 100% success in the first condition of the LB procedure under ND, eighteen of them (36%) (ten male, eight female) had success with the PF procedure, and finally seven participants (14%) (two male, five female) had success with the AL procedure. The results showed that none of the fifty participants had a successful prediction with all three procedures (ND, PF, AL) of the LB part of the experiment. In fact, twenty-three students (46%) (ten males, thirteen females) did not give a correct answer for any of the procedures of the LB part.

We also examined the severity of errors in the students’ predictions. The tabs were ordered according to the value property of the color, i.e., the order of the tabs in the VITA classical shade guide was as follows: B1, A1, B2, D2, A2, C1, C2, D4, A3, D3, B3, A3.5, B4, C3, A4, and C4. Then, we translated these codes into a sequence of numbers from 1 to 16, starting with 1 for the lightest color (B1) and 16 for the darkest color (C4). In this way, we were able to translate how closely the participants’ predictions matched the actual shade of the tab. Table 2 shows the percentages of predictions and the deviations from the actual predictions for each of the three conditions in the LB part of the experiment. The results show that 10% of the participants in the ND condition were one shade and 15% were two shades away from the correct prediction in either direction of the shade guide. Similarly, 15% and 10% were one or two steps away from the actual prediction when using the PF. Further, 15% were one or two shades off when using AL. These subtle differences would bring the correct predictions for all conditions to over 70%. The most common errors detected one step away from the correct prediction were A1–B1, A3–D3, A3.5–B4, and A4–C4.

#### 3.1.2. Clinical (CL) Phase: Natural Tooth Shade Selection

The students’ performance decreased in the CL phase compared to the LB phase. In the CL phase, the overall success of the students was 32%, compared to the LB phase (overall success: 43–52%). The shade matching between males and females was as follows: In ND, male participants had 28% correct predictions and female participants had 36% correct predictions. In AL, the result was the opposite: 28% of female participants and 36% of male participants chose the correct color. Finally, an equal percentage of both sexes (32%) gave a correct answer when using the PF. Accordingly, the female participants seemed to perform better on the CL test under ND (36%), followed by the use of the PF (32%), and finally AL (28%). The male participants seemed to have achieved their best performance using the light aids. The highest success rate of 36% was found when using AL, followed by the PL (32%), and finally ND (28%). The McNemar test was used to compare correct predictions in the CL condition between male and female students, and this showed that there were no significant differences in correct predictions in the CL condition between male and female students, at a 5% level of significance (Table 1).

Of the fifty participants, a total of eight participants (16%), five male and three female, found the correct color using all three methods (ND, PF, AL) of the CL procedure. A surprising number of 29 participants (58%), 15 males and 14 females, did not give a correct answer in any of the methods of the CL session.

When the deviation of error was examined, the results were as follows: 28% of participants were one step away from the correct prediction and 8% were two shades away from the correct prediction when it came to ND. Similarly, 26% and 8% were one or two steps away from the actual prediction when the PF was used. When AL was used, 24% were off by one shade and 14% were off by two shades. These subtle differences would increase the correct predictions to about 70% for all conditions. Table 2 shows the percentages of predictions and the deviations from the actual predictions for each of the three conditions in the LB part of the experiment.

#### 3.1.3. Comparisons Between CL and LB Male and Female Results

To compare the deviation of the participants’ prediction with the actual tab shade of the LB experiment or the natural tooth color of the CL experiment, we performed an independent samples t-test. This test was also performed to compare possible differences in the mean absolute difference between male and female students. The basic results of this test are shown in Table 3. The smallest difference was found in the female participants when the PF was used in the LB phase (mean: 1.14, SD: 1.65) and the largest difference was found in the male participants when the PF was used in the CL phase (mean: 2.28, SD: 2.64). However, there was no statistically significant difference between males and females in any of the six conditions at a 5% significance level (5), so there are no sex differences in the prediction of color shades.

We also examined the number of participants who were 100% successful with their predictions in both parts of the experiment (LB and CL) under the three light conditions (ND, Al, PF) (Table 4). The results of the ND condition show that five participants had correct predictions in both the LB experiment and the CL experiment. This corresponds to a 10% rate of participants with absolute success. In contrast, 22% of the participants had an absolute failure in that they did not achieve a correct answer for any prediction under ND. Accordingly, the absolute success of the participants’ predictions under PF was 8% and the absolute failure was 20%. In the corresponding results for the AL conditions, only 2% of participants were able to make a correct prediction under both the LB and CL phases, while 18% of participants did not give a correct answer.

### 3.2. Qualitative Results

#### Response to Questionnaire

After completing the experiment in the LB and CL settings, the participants were asked to answer a questionnaire generated via QR code (Figure 1). The first question related to the participants’ preference between the conditions of this experimental procedure, i.e., the ND, AL, and PF as well as the spectrophotometer. As can be seen, 58% of the participants opted to use a spectrophotometer for color selection, as they felt that the results of the instrument were more reliable for color selection, followed by color selection under ND (36%), and finally the use of lighting aids (AL + PF) (8%). When participants were asked about the simplicity of the methods, the vast majority of participants chose to use the spectrophotometer (86%) as it was a quicker method, and the results were automatically displayed on the screen of the device with just one touch of a button. Impressively, none of the participants chose to use light aids (Al/PF), while the remaining students chose the shade selection under ND (14%).

When the participants were asked whether the lighting aids (AL/PF) were beneficial for shade selection compared to ND, 64% of the students responded positively, although they previously indicated that shade selection with lighting aids was not an easy method (Question 2). The participants were informed about the cost of the equipment (AL, PF, and spectrophotometer) and then asked whether they would invest in the equipment for their own practice in the future. It appears that three out of four participants were willing to purchase the devices in the future.

**Figure 4:**
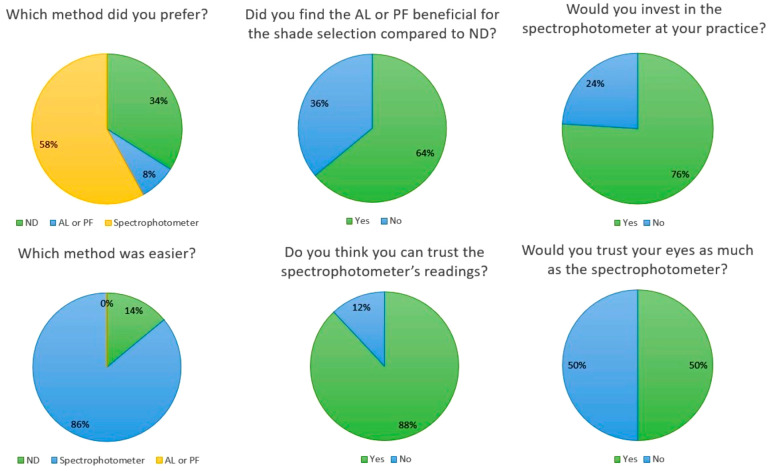
Pie charts of the answers of participants to the QR code-generated questionnaire.

The next question was related to the level of confidence that the participants felt and how much they trusted their own eyes without a light aid or spectrophotometer. Half of the students responded that they would trust their eyes as much as the spectrophotometer when selecting tooth color, while 12% of the participants answered that they did not trust the readings of the spectrophotometer. A schematic representation of these answers is visualized in Figure 4.

## 4. Discussion

The study aimed to evaluate the ability of dental students to accurately match dental shades under different lighting conditions using various lighting aids and shade selection tools. Overall, the findings reveal that students were more successful in shade matching under natural daylight (ND) and when using a polarizing filter (PF) compared to artificial light (AL). The success rates were 52% for the PF, 50% for ND, and 43% for AL in the laboratory (LB) setting. In the clinical (CL) setting, success rates dropped, with 32% of correct predictions overall. This suggests that environmental factors and the complexity of working with natural teeth in vivo impact shade selection accuracy, as seen in the reduced success rates in the clinical setting. None of the results reached the level of statistical significance, thus rejecting the first hypothesis that light aids would be more accurate in the process of shade selection by candidates.

The study also highlights the relatively small differences between male and female participants across all conditions, with no statistically significant difference found in color-matching ability between sexes. This result leads to the acceptance of the second hypothesis as sex was not an important factor in shade matching. In fact, based on the results of this study, there was no statistically significant difference between participants of different biological sexes, in any setting (laboratory or clinical), neither with nor without the use of light aids. This is in line with the results of Dudea et al., where there was no statistically significant difference in terms of sex and lighting conditions for the purpose of shade matching [10]. There are a few studies that have suggested that females are better at shade matching [4,24] and other studies that have shown no significant influence of sex on tooth shade selection [12,25]. Actually, the literature suggests that even the same participant may not choose the same shade in two separate sessions and that a successful prediction is experience- and occupation-dependent [25].

Color matching, a critical step in prosthetic and restorative dentistry, relies on shade-taking devices as the gold standard for cost-effective tooth color assessment, with spectrophotometric methods offering superior precision, reproducibility, and enhanced communication for dental restorations, though they require proper use and complementary color mapping to ensure seamless blending with adjacent teeth [6,11].

The VITA classical shade guide was utilized in this study because, despite its flaws, it is still regarded as “the gold standard.” Another reason for choosing VITA classical over VITA 3D-MASTER ^®^, which has been demonstrated to be a better shade guide, was that the former version featured fewer tabs (16 vs. 29). The time required to match 29 tabs in a single session would probably have reduced the number of accurate answers due to eye fatigue [10].

The findings align with the existing literature, which suggests that natural daylight remains a reliable source for shade matching in dentistry. The slight improvement with the polarizing filter in this study corroborates findings that using filtered light can reduce glare and reflections, aiding in more accurate color matching. However, the relatively low success rate under artificial light (AL) mirrors findings from earlier studies which argue that ambient and artificial light sources can distort shade perceptions [11]. The reliance on a spectrophotometer for validation in the in vivo part of this study is consistent with other studies showing that instrumental shade matching tends to provide more consistent and objective results compared to subjective visual matching [11]. In fact, the color difference (ΔΕ) of spectrophotometric measurements was found to be 0.48, whereas the smallest color difference the human eye can detect is ΔΕ of 1.0 under standard conditions, but this reaches ΔΕ of 3.7 in intraoral conditions [17]. The preference of participants for the spectrophotometer, despite expressing confidence in their visual skills, indicates the device’s practical advantages in clinical settings where accuracy is crucial.

In clinical practice, achieving an accurate shade match is critical for esthetic restorative outcomes. This study demonstrates that there are considerable difficulties in shade matching accuracy, especially in the clinical setting, suggesting that replicating controlled conditions (e.g., consistent lighting, use of shade guides) is crucial for improved outcomes in patient care.

Some of the parameters in the experimental set up were defined by findings in the literature and previous publications. The participants were confined to undergraduate fourth-year dental students who had a previous theoretical background in shade selection but with limited clinical experience as the question was to seek the ability of this group to select the correct shade. It is known that the level of expertise and profession may affect this ability, as found in studies involving restorative dentists, general dentists, dental technicians, dental assistants, and students [26]. None of the participants had sight deficiencies as revealed by the PIP test, although in the literature it is common to find these discrepancies, especially in male participants, and this partly explains why it is a common belief that females do better at shade selection procedures. The VITA classical shade guide was chosen for its ease of use and having fewer possible shade options, although it was known that there were other shade guides with more standard shade differences between adjacent shade tabs than the selected shade guide (e.g., the 3D-MASTER^®^, VITA) [17]. The gray background was selected as it was found to have fewer distorting results in other studies [2,13]. The shade tabs were arranged according to the hue in the unblinded shade chart [3,7].

The last step of the examination was to carry out shade matching using a spectrophotometer (VITA EasyShade). This measurement served as a comparative shade of the examined natural tooth. The measured tooth shade was accepted when three consecutive identical readings were obtained for each tooth [20]. So far, there is limited scientific evidence comparing the effectiveness of different shade-matching devices and for this reason, the spectrophotometer had an auxiliary role in the research. It was used to familiarize students with advanced technological tools and provide them with a different method of shade selection. In fact, students are an ideal group for evaluating shade-matching abilities, as they are typically young adults within a similar age range, have limited prior experience with shade selection, and are less likely to have systemic conditions affecting color perception [5,9]. As shown from the responses to the questionnaire, the participants gained confidence by using the spectrophotometer, thus this auxiliary tool could enhance their ability to accurately determine tooth shade in the isolated environment of their own practice during their first professional steps.

The finding that no participant achieved 100% success across all conditions underscores the challenges of visual shade matching and the potential need for additional tools, such as the spectrophotometer, to complement visual assessments. The high percentage of students willing to invest in advanced shade-matching technology reflects an awareness of the limitations of subjective methods and a desire to improve clinical outcomes.

### 4.1. Limitations of the Study

One limitation of the study is the controlled environment of the dental clinic, which may not fully reflect the variations in lighting that can occur in real-world clinical settings. Additionally, the relatively small sample size (*n* = 50) may limit the generalizability of the results to broader populations of dental professionals. Future studies could expand the sample size and include participants with varying levels of experience to see how shade-matching accuracy correlates with expertise. The VITA EasyShade (ES) uses spot measurements, which may face accuracy challenges due to tooth shade variability, surface curvature, and drying effects. A whole-tooth measuring device with topographical color mapping (e.g., Spectroshade Micro SS) could be more accurate as a control [27,28,29].

### 4.2. Future Research Directions

Further research could explore the impact of advanced training in shade matching on students’ performance, as this study did not provide additional training prior to the experiment. Another avenue for future research is to investigate how other variables, such as tooth hydration or patient complexion, influence shade matching under different lighting conditions. Additionally, incorporating advanced color-matching technologies (such as shade matching by intraoral scanners) and comparing them to both visual and spectrophotometric methods could yield insights into the most effective strategies for achieving optimal restorative outcomes. Future research on the efficacy of various shade-matching devices could provide deeper insights into their accuracy. Integrating these devices into clinical training at universities would be advantageous for assessing their potential as educational tools.

## 5. Conclusions

This study emphasizes the importance of proper lighting conditions and tools in dental shade matching. While natural daylight and polarizing filters proved effective, the clinical setting posed additional challenges. Spectrophotometers offer a promising solution for more reliable shade selection, and the willingness of students to invest in such technology reflects its value in modern dental practice. However, ongoing education and exposure to different shade-matching techniques remain essential for improving the accuracy of dental restorations.

## Figures and Tables

**Table 1:** Cross tabulation of the correct shade prediction (LB: lab, CL: clinical, ND: natural daylight, PF: polarized filter, AL: artificial light).

Cross Tabulation of the Correct Shade Prediction of the Lab (LB) Part							
		Male		Female		Total	
		n	Percentage	n	Percentage	n	Percentage
Correct Score under natural daylight (ND)	False	25	50.0%	25	50.0%	50	50.0%
	Correct	25	50.0%	25	50.0%	50	50.0%
	Total	50	100.0%	50	100.0%	100	100.0%
Correct Score under polarized filter (PF)	False	24	48.0%	24	48.0%	48	48.0%
	Correct	26	52.0%	26	52.0%	52	52.0%
	Total	50	100.0%	50	100.0%	100	100.0%
Correct Score under artificial light (AL)	False	29	58.0%	28	56.0%	57	57.0%
	Correct	21	42.0%	22	44.0%	43	43.0%
	Total	50	100.0%	50	100.0%	100	100.0%
Cross Tabulation of the Correct Shade Prediction of the Clinical (CL) Part							
		Male		Female		Total	
		n	Percentage	n	Percentage	n	Percentage
Correct Score under natural daylight (ND)	False	18	72.0%	16	64.0%	34	68.0%
	Correct	7	28.0%	9	36.0%	16	32.0%
	Total	25	100.0%	25	100.0%	50	100.0%
Correct Score under polarized filter (PF)	False	17	68.0%	17	68.0%	34	68.0%
	Correct	8	32.0%	8	32.0%	16	32.0%
	Total	25	100.0%	25	100.0%	50	100.0%
Correct Score under artificial light (AL)	False	16	64.0%	18	72.0%	34	68.0%
	Correct	9	36.0%	7	28.0%	16	32.0%
	Total	25	100.0%	25	100.0%	50	100.0%

**Table 2:** Shade difference between actual tab shade and students’ prediction under three conditions (LB: lab, CL: clinical, ND: natural daylight, PF: polarized filter, AL: artificial light).

Shade Difference Between Actual Tab Shade and Students’ Prediction LB Part			
Absolute Difference	Natural Daylight	Polarized Filter	Artificial Light
0	50	52	43
1	10	15	15
2	15	10	15
3	5	8	8
4	7	9	11
5	8	4	3
6	2	2	3
7	1	0	2
8	1	0	0
10	1	0	0
Total	100	100	100
Shade Difference Between Actual Tab Shade and Students’ Prediction Clinical (CL) Part			
Absolute Difference	Natural daylight	Polarized filter	Artificial light
0	16	16	16
1	14	13	12
2	4	4	7
3	7	9	8
4	2	3	2
5	3	4	5
6	3	0	0
9	1	0	0
12	0	1	0
Total	50	50	50

**Table 3:** Deviation of the participants’ prediction with the actual shade tab for male and female students under several conditions (LB: lab, CL: clinical, ND: natural daylight, PF: polarized filter, AL: artificial light).

	Male			Female			t	d.f.	*p*
	n	Mean	S.D.	n	Mean	S.D.			
LB–ND	50	1.66	2.08	50	1.54	2.23	0.28	98	0.781
LB–PF	50	1.40	1.73	50	1.14	1.65	0.77	98	0.444
LB–AL	50	1.66	1.94	50	1.54	1.82	0.32	98	0.751
CL–ND	25	1.96	2.15	25	1.76	2.09	0.33	48	0.740
CL–PF	25	2.28	2.64	25	1.40	1.53	1.44	48	0.155
CL–AL	25	1.64	1.63	25	1.68	1.68	−0.09	48	0.932

**Table 4:** Crosstabulation of participants’ shade predictions under the three light conditions (ND: natural daylight, PF: polarized filter, AL: artificial light) in both **CL** (clinical) and **LB** (lab) settings.

		Correct Score LB and ND			Total
		False Both	One Correct	Two Correct	
Correct Score CL and ND	False	11	13	10	34
	Correct	4	7	5	16
Total		15	20	15	50
		Correct Score LB and PF			Total
		False both	One Correct	Two Correct	
Correct Score CL and PF	False	10	10	14	34
	Correct	6	6	4	16
Total		16	16	18	50
		Correct Score LB and AL			Total
		False	One Correct	Two Correct	
Correct Score CL and AL	False	9	19	6	34
	Correct	5	10	1	16
Total		14	29	7	50

## Data Availability

The original contributions presented in this study are included in the article. Further inquiries can be directed to the corresponding author.

## Abbreviations

The following abbreviations are used in this manuscript:

LB	Laboratory settings
CL	Clinical settings
ND	Natural daylight
AL	Artificial light
PF	Polarized filter
